# 

**Published:** 1907-10-12

**Authors:** 


*NOW READY.*

### NERVE DISEASES FOR STUDENTS.

By L. A. CLUTTERBUCK, M.D., B.S. (Durham), M.R.C.P. (London), L.R.C.P. & S. (Edin.), L.F.P. & S. (Glasg.), L.R.C.P. (Ireland); Hon. Physician, St. Marylebone General Dispensary; Clinical Assistant, West End Hospital for Nervous Diseases, London, &c.

**Price 3s. net, by post 3s. 3d.**

Of all Booksellers, or of the Publishers—
THE SCIENTIFIC PRESS, LTD.,
28/29 Southampton Street, Strand, London, W.C.

